# In Vitro Assessment of Postbiotic and Probiotic Commercial Dietary Supplements Recommended for Counteracting Intestinal Dysbiosis in Dogs

**DOI:** 10.3390/vetsci11010019

**Published:** 2024-01-03

**Authors:** Benedetta Belà, Maria Magdalena Coman, Maria Cristina Verdenelli, Alessandro Gramenzi, Giulia Pignataro, Dennis Fiorini, Stefania Silvi

**Affiliations:** 1 Department of Science of Veterinary Medicine Science, Public Health and Animal Wellness, University of Teramo, Via R. Balzarini 1, 64100 Teramo, Italy; platobio31@gmail.com (B.B.); agramenzi@unite.it (A.G.); giuliapignataro@gmail.com (G.P.); 2 Synbiotec Srl spin-off di UNICAM, Via Gentile III da Varano, 62032 Camerino, Italy; magda.coman@unicam.it (M.M.C.); cristina.verdenelli@unicam.it (M.C.V.); 3 School of Science and Technology, Chemistry Division, University of Camerino, Via Madonna delle Carceri 9/B, 62032 Camerino, Italy; dennis.fiorini@unicam.it; 4 School of Biosciences and Veterinary Medicine, University of Camerino, Via Gentile III da Varano, 62032 Camerino, Italy

**Keywords:** dietary supplements, probiotics, postbiotics, dogs, microbiota, short-chain fatty acids

## Abstract

**Simple Summary:**

The authors evaluated the effects of two specific commercial dietary supplements: a combination of a postbiotic and prebiotics (Microbiotal cane^®^—NBF Lanes S.r.l., Milan, Italy) and a probiotic product (NBF 1^®^), recommended for counteracting intestinal dysbiosis in dogs, on the gut canine microbiota composition and its metabolic activities (production of short-chain fatty acids). The investigation was performed using an in vitro fermentation system inoculated with dog fecal samples; both of the dietary supplements significantly modulated the dog intestinal microbiota. Microbiotal cane^®^ manages to have a short-term effect, increasing the concentration of lactobacilli, whereas NBF 1^®^ may be of benefit in reducing excessive proliferation of bacterial species, like *Bacteroides–Prevotella–Porphyromonas* spp. In case of alterations in the intestinal microbiota, the two products can be used selectively by modulating the growth/decrease of specific bacterial species.

**Abstract:**

Many environmental aspects influence the preservation of a beneficial microbiome in dogs, and gut dysbiosis occurs when imbalances in the intestinal ecosystem cause functional changes in the microbial populations. The authors evaluated the effects of two specific commercial dietary supplements: a combination of a postbiotic and prebiotics (Microbiotal cane^®^) and a probiotic product (NBF 1^®^) recommended for counteracting intestinal dysbiosis in dogs, on the gut canine microbiota composition and its metabolic activities (production of short-chain fatty acids). The investigation was performed using an in vitro fermentation system inoculated with dog fecal samples. Microbiotal cane^®^ promoted a more immediate increase in *Lactobacillus* spp. after the first 6 h of fermentation, whereas NBF 1^®^ promoted the increase at the end of the process only. The two supplements supported an increase in the *Bifidobacterium* spp. counts only after 24 h. The in vitro abilities of Microbiotal cane^®^ and NBF 1^®^ to increase selectively beneficial bacterial groups producing acetic, propionic, and butyric acids suggest a possible positive effect on the canine gut microbiota, even if further in vivo studies are needed to confirm the beneficial effects on the intestinal health.

## 1. Introduction

Dogs, like cats, belong to the order of carnivores, so their main source of nourishment is made up of animal tissues. However, comparative studies of the nutritional needs, anatomical characteristics, and metabolic adaptations of these two species show how differently they have evolved from each other.

During evolutionary development, cats have always been obligated carnivores, but dogs, instead, are defined as opportunistic carnivores, developing eating habits more similar to those of omnivores. This is because dogs have lived in greater contact with man, gradually adapting to his type of diet. Most canine microbiome studies [1,2] looked to extruded kibble, representing up to 95% of the dry dog food market [3]. Traditionally, the extrusion process requires a high content of carbohydrates (at least 30% starch in % dry matter—DM) in the form of vegetable ingredients. Recently, new alternative industrial processes allow the inclusion in the pet food market of reduced carbohydrate and increased protein-content kibble (no more than 15% starch in % DM and at least 25% protein in % DM) [3].

It is well known that high-protein vs. high-carbohydrate diets generate different gut microbiome profiles. The maintenance of a healthy microbiome is affected by many other environmental factors; however, the changes they cause have less impact when compared to the alterations found in diseased animals. GI dysfunctions are mainly associated with gut dysbiosis. Acute and chronic diarrhea, obesity, metabolic diseases, cancer, and neurological dysfunctions have been associated with gut microbiome modifications [3]. Similar to studies on healthy dogs, those on dogs with GI diseases also reported different taxa abundance percentages [4]; however, most taxa consistently vary within the same disease phenotype.

Manipulations of the microbiome are often part of the treatment; several strategies have been proposed to improve and maintain the equilibrium of the intestinal microbiota, including probiotic supplementations. Currently, probiotics are also used to prevent or minimize symptoms due to the use of anti-inflammatory treatment [5], acting on the frequency of diarrhea, the composition of the fecal microbiota, and/or markers of GI inflammation. Sometimes, the principal disadvantage of a probiotic supplementation is their low persistence in the canine gastrointestinal tract after finishing the probiotic supplementation [6,7]. A further approach is the use of synbiotics, in which the presence of prebiotic ingredients should support probiotic growth, increasing its proliferation within the GI tract and offering its own advantages [8,9]. However, the use of a combination of postbiotic and prebiotic supplementations in the canine diet has not yet been adequately explored.

The term postbiotic is derived from ‘biotic’, defined as ‘relating to or resulting from living organisms’, and ‘post’, a prefix meaning ‘after’; together, these terms suggest ‘after life’, that is, non-living organisms; postbiotic is a ‘preparation of inanimate microorganisms and/or their components that confer a health benefit on the host’ [10]. Although the effects of postbiotics on the microbiota might be temporary, they can have direct and indirect antimicrobial activities. With regards to the direct antimicrobial activity, in vitro studies recognize compounds, such as bacteriocins, that have an antimicrobial action; on the other end, the indirect antimicrobial activity is associated with the transport of quorum-sensing (favoring the microorganisms communication) and quorum-quenching (capable of interrupting communication) molecules. Moreover, other studies highlighted the importance of other molecules like lactic acid that can be consumed by some members of the intestinal microbiota, resulting in SCFAs and butyrate production, which have beneficial functions [11].

Postbiotics also perform local and systemic immunomodulatory functions by activating an immune response to fight infections and reduce the infection degree in the acute phase; some of the immunomodulatory microbial metabolites are branched-chain fatty acids and SCFAs. In addition, they improve the epithelial barrier functions; from *Bifidobacterium* spp. is derived substances promoting barrier function, reducing inflammation with mechanisms under study.

SCFAs found in postbiotics also have the potential to modify the function of the epithelial barrier, helping to keep it intact; they serve to increase transepithelial resistance and to stimulate the formation of the tight junction in some of the intestinal epithelial cells. Succinate, a bacterial intermediate in carbohydrate fermentation as well as a substrate for intestinal gluconeogenesis, appears to act on glycemic control, improving systemic metabolism.

Other research has also shown how postbiotics can modulate cognitive function and behavior using different neuroactive compounds, including neurotransmitters, such as serotonin, dopamine, acetylcholine, GABA, and various compounds that can bind to receptors expressed in the brain, such as serotonin. While the study of pre- and probiotics began several years ago, postbiotics have only recently entered the heart of scientific research; currently there are few works, especially in vivo studies, that have examined the effect of postbiotics and their possible combinations on the composition of the intestinal microbiota, representing another therapeutic strategy for modulating the gut microbiota of healthy and/or diseased animals.

In the present study, the authors evaluated whether two specific commercial dietary supplements (a combination of a postbiotic and prebiotics and a probiotic product) recommended for counteracting intestinal dysbiosis in dogs affected the quantity, composition, and metabolic activities (production of SCFAs) of canine gut microbiota after 6 h and 24 h of supplementation, compared to time zero.

The investigation was performed using an in vitro fermentation model simulating the dog intestinal tract. The in vitro model, also utilized in previous studies [12,13], offer several advantages, including dynamic sampling over time and high reproducibility, without the ethical issues that can arise in clinical human contexts. 

## 2. Materials and Methods

### 2.1. Commercial Products Tested

Two commercial dietary supplements dedicated to counteracting intestinal dysbiosis in dogs were tested. Microbiotal cane^®^ (NBF Lanes S.r.l., Milan, Italy) are tablets with a purplish color, are odorless, and contain tindalized *Limosilactobacillus reuteri* NBF 1 (formerly *Lactobacillus reuteri*) DSM 32203 [12], fructo-oligosaccharides (FOS), chicory inulin, microencapsulated butyric acid, and polyphenols from blood orange. NBF 1^®^ (NBF Lanes S.r.l., Milan, Italy) is a white powder, is odorless, and consists of the live probiotic bacterial strain *L. reuteri* NBF 1 (formerly *Lactobacillus reuteri*) DSM 32203 [14].

### 2.2. Batch Culture Fermentations

A pilot fermenter (Applikon Fermentation System, Applikon Biotechnology, Delft, The Netherlands), stirred and pH- and temperature-controlled, was used to perform the anaerobic batch culture. The whole procedure was previously described by Belà et al. [12].

The inoculum was prepared using fecal samples obtained from 3 adult healthy dogs following the procedure reported by Belà et al. [12]. Subjects were fed a standard diet free of components that could influence the analysis; they had not consumed any antibiotics for at least one month before the study, had no history of GI disease, and were not regular consumers of probiotic/prebiotic supplements. The fecal samples were collected by the owners of the dogs, who gave their consent to offer the already naturally deposited stools. Ethical review and approval were waived for studies in which protocol requirement for the administration of a nutraceutical is covered by Directive 2010/63/EU of the European Parliament and by the Council of 22 September 2010 on the protection of animals used for scientific purposes.

Fecal slurry (1%) was the inoculum of each batch culture fermentation. The latter were singularly supplemented and named as follows: Microbiotal was added with 0.5% (5 g) of the Microbiotal cane^®^ supplement, and NBF 1 had, as inoculum, 10^9^ CFU/mL of the NBF 1^®^ supplement. The supplement concentration simulated the real dose recommended for use by the manufacturer. 

The day before fermentation, the product chosen to be fermented and the fecal inoculum (fecal sample previously prepared and stored at −80 °C) were transferred from −80 °C into the cold room, while on the same day of fermentation, the saline solution, hemin, vitamin K, bile acids [12], and the product to be tested were added to the medium just before starting fermentation. 

The pH was adjusted at 6.80 ± 0.2 before inoculating the medium. The system was permanently gasified with N_2_ fed at 15 mL/min. The anaerobiosis, the temperature of 39 ± 1 °C, and the stirring (at 50/55 rpm) were kept for a period of 24 h, simulating the conditions of the canine intestine. During the fermentation, samples’ aliquots of 10 mL were collected at three specific time points: T0 (the beginning of fermentation), T6 (after 6 h of fermentation), and T24 h (after 24 h of fermentation). This latter time point was included to better understand the long-term fermentation changes on the microbiota, considering the model over a realistic time. Samples were stored at −20 °C and higher until be analyzed for bacterial enumeration and quantification of short-chain fatty acids.

### 2.3. Selective Enumeration of Bacteria Groups, Bacterial Profiling of the Fluid Microbiota, and SCFAs Determination

At each time point, two fermented fluid aliquots were used for bacterial enumeration/profiling and for SCFA quantification, respectively.

Real-time PCR was performed for the enumeration of selected bacterial groups, as described by Belà et al. [12], after extraction of bacterial DNA [15] from one aliquot of fermented fluid. The bacterial groups of interest were *Bifidobacterium* spp., *Lactobacillus* spp., *Bacteroides–Prevotella–Porphyromonas* spp., *Staphylococcus* spp., *Clostridium coccoides–Eubacterium rectale* group, and Enterobacteriaceae. SYBR Green real-time PCR amplifications were performed using an iCycler iQ real-time detection system (Stratagene, La Jolla, CA, USA) associated with the MXP Software (2.0 version). All PCR experiments were carried out in triplicate, with a reaction volume of 20.6 μL, using iCycler IQ 96-well optical grade PCR tubes (Stratagene) covered with an iCycler optical cap (Stratagene).

The efficiency of PCR amplification was optimized with a primer concentration of 500 ng/μL (pmol/μL), which was proved to be optimal for the amplifications of target sequences. The reaction mixtures contained 9.8 μL of iTaqUniversalSYBR Green Supermix (Bio-Basic, Amherst, NY, USA), 9 μL of distilled sterile water, and 0.4 μL of each primer (forward and reverse, DSMZ). Subsequently, 1 μL of DNA (or water for negative control) was added to the reaction mixture.

For each bacterial strain, the total bacterial copy number per organism was determined with 16S rRNA gene-targeted primers.

Amplification was performed with the initial temperature at 95 °C for 2 min. UniversalSYBR probe activation, 35–40 cycles of denaturation at 95 °C for 15–30 s, annealing temperature kept at the optimal temperatures for 20–60 s, extension at 72 °C for 30 s, and an additional incubation step at the same annealing temperature and length were performed to collect fluorescent data [12,16]. 

In addition, from the extracted DNA, high-resolution genotyping of the fermentation fluid microbiota was performed to comprehensively characterize the microbial composition of the fluid during that time. The 16S metagenomic analysis was carried out using a 16S next-generation sequencing (NGS) approach, as described by Micioni Di Bonaventura and colleagues [17].

A second aliquot of fermentation fluid was used for the extraction and quantification of SCFAs, following the analytical procedure reported by Scortichini et al. [18].

### 2.4. Statistical Methodology

The experimental fermentations were conducted in duplicate, and the analysis was repeated in duplicate. The data were expressed as the mean value ± standard deviation. One-way ANOVA and Tukey’s multiple comparison test were used to compare different time points within the same fermentation experiment and the two kinds of fermentations.

For the 16S NGS data, differences in relative abundances of taxa between the two types of fermentations were evaluated using the four multiple comparison test (R version 4.2.1), with a significance level of *p* < 0.05. 

## 3. Results

### 3.1. Bacterial Populations Changes

**Figure 1:**
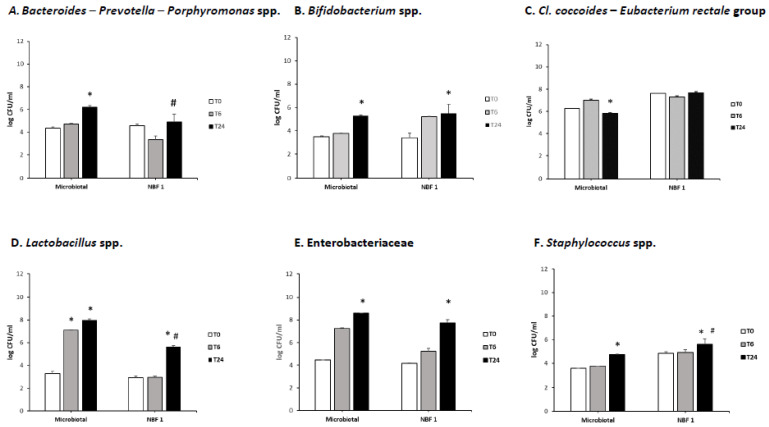
Bacterial counts (log CFU/mL) of *Bacteroides–Prevotella–Porphyromonas* spp. (**A**), *Bifidobacterium* spp. (**B**), the *Clostridium coccoides–Eubacterium rectale* group (**C**), *Lactobacillus* spp. (**D**), Enterobacteriaceae (**E**), and *Staphylococcus* spp. (**F**) detected at different time points of fermentation. Significantly different (*p* < 0.05 by one-way ANOVA and Tukey’s multiple comparison test): * vs. T0 inside the same fermentation sample; # vs. Microbiotal fermentation.

Figure 1A–F reports the counts of the selected bacterial groups that were detected in each fluid of the two supplemented fermentations at the T0, T6, and T24 time points. The *Bacteroides–Prevotella–Porphyromonas* spp. count showed a different trend during the fermentation of the two supplements: the Microbiotal fermentation presented a slight increase in the amount of this bacterial group immediately after 6 h (T6), and a significant increase of about 2 logs was detected at the end of the fermentative process (T24) (Figure 1A). On the contrary, the NBF 1 fermentation promoted a slight decrease in that bacterial group after 6 h (T6), as compared to the starting point, followed by a considerable increase at the end of the fermentation (T24), even if not statistically different vs. T0 (Figure 1A). *Bifidobacterium* spp. displayed quite a similar trend during the two product fermentations: after 6 h (T6), there was a very slight increase, as compared to the starting point (T0), while at the end of the process (T24), there was an appreciable increase of about 2 logs in the count of this bacterial group, as compared to the starting point (T0) (Figure 1B). The *Cl. coccoides*–*Eu. rectale* group tended to increase after 6 h (T6) of Microbiotal fermentation and decreased at the end of the process (T24) (Figure 1C). Meanwhile, during the fermentation with NBF 1, the amount of this bacterial group remained quite stable, with a very slight decrease after 6 h (T6), as compared to the beginning (T0) (Figure 1C). The *Lactobacillus* spp. count showed a marked increasing trend during the fermentation of Microbiotal cane^®^: after 6 h (T6) the count of this bacterial group increased by about four logs, as compared to the initial time point (T0) (Figure 1D), with a further increase at the end of the process (T24) of one more log (Figure 1D). The NBF 1 fermentation caused a significant increase in the *Lactobacillus* spp. count only at the end of the process (T24), as compared to the starting point (T0) (Figure 1D). The Enterobacteriaceae count displayed a comparable increasing trend during the fermentation of the two products; it was more pronounced during Microbiotal fermentation: after 6 h (T6), this bacterial group increased by about 3 logs, as compared to the starting point (T0) (Figure 1E), reaching a final count of 4.0 × 10^8^ CFU/mL at the end of the process (T24) (Figure 1E). The fermentation with NBF 1 showed a lower increase in the amount of Enterobacteriaceae after 6 h, but then it also reached a significant level, as compared to the initial amount (Figure 1E). Finally, both the product fermentations promoted an increase in the *Staphylococcus* spp. at the end of the process (T24) (Figure 1F), compared to the beginning (T0) (*p* < 0.05), and NBF 1 significantly stimulated the staphylococci growth, compared to Microbiotal.

### 3.2. Profiling of the Fluid Microbiota by 16S Next-Generation Sequencing (NGS)

**Figure 2:**
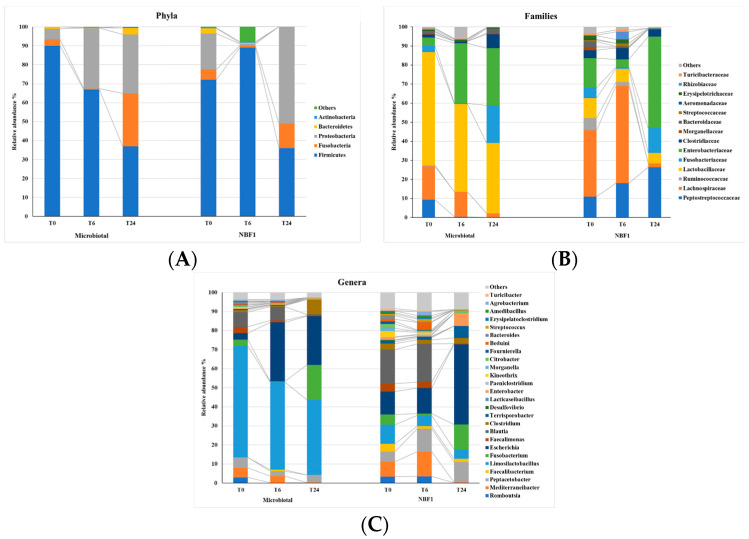
Relative abundances of the phyla (**A**), families (**B**), and the top 26 genera (**C**). “Others” include other species. The x-axis represents time points (T0, T6, and T24), and the y-axis represents the relative abundance percentage.

The composition of microbiota developed in the two fermentation fluids was profiled during three time points: T0, T6, and T24. The change in α-diversity in the Microbiotal and NBF 1 fermentation fluids was evaluated by the Shannon, Simpson, chao1, and adv indices. No significant difference in terms of species richness was observed (*p* > 0.05). The Bray–Curtis distances used to reveal β-diversity, that is, bacterial structural differences, of the two fermentations did not show a congruent directional pattern between the two groups of data. Analysis of the taxonomic composition at T0 revealed the presence of five major bacterial phyla: Firmicutes, Fusobacteria, Proteobacteria, Bacteroidetes, and Actinobacteria in both the starting-point fermentation fluids; among these, Firmicutes, Fusobacteria, and Proteobacteria were the most abundant members (Figure 2). 

Taxonomic variation trends were detected within the two fermentations from T0 to T24, with a decrease in Firmicutes, though not significantly (*p* > 0.05), and concomitant increases in Fusobacteria and Proteobacteria (the last in a significant way, *p* < 0.05).

At the family and genus levels, 16 families and 26 genera were the dominants in both fermentation fluids (relative abundance of >1%; Figure 2).

The Microbiotal fermentation fluid showed an initial presence of Lactobacillaceae (59%) with the major presence of *Limosilactobacillus reuteri* (58%)—as expected by the composition of the commercial product (tindalized *L. reuteri*)—slightly decreasing at 24 h coincident with the T24-value of *L. reuteri*. In the NBF 1, the *L. reuteri* was detected as 10% at T0—supplied by the live strain product—and moderately decreased at 24 h of fermentation. In both fermentations, the Fusobateriaceae, present at a low level at T0, reached, at T24, a higher abundance in Microbiotal fermentation and was completely represented by the *Fusobacterium* genus. A further significant increase (*p* < 0.05) was related to the abundances of Enterobacteriaceae that reached 30% and 48%, respectively, in Microbiotal and in NBF 1 fermentations; the *Escherichia* genus was the most represented.

### 3.3. SCFA Levels

**Figure 3:**
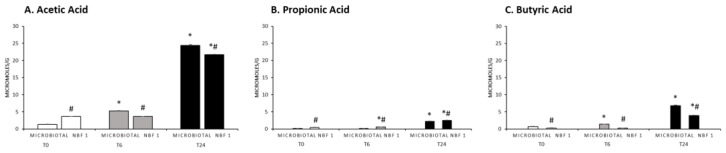
Concentrations (µmol/g) of acetic acid (**A**), propionic acid (**B**), and butyric acid (**C**) in the fermentation fluid samples of each tested product at the three time points of the fermentative process. Significantly different (*p* < 0.05 by one-way ANOVA and Tukey’s multiple comparison test): * vs. T0 inside the same fermentation sample; # vs. Microbiotal fermentation.

The main SCFAs (acetic, propionic, and butyric acids, expressed in µmol/g) detected during the fermentation of the tested products are reported in Figure 3A–C. Generally, they reached significantly higher levels at T24 than T0. Acetic acid was the one that increased more during the fermentations of both products, especially at the end of the process (T24). Microbiotal fermentation caused a significantly higher increase with respect to the NBF 1 fermentation (Figure 3A). The amount of propionic acid increased very slightly during the fermentations of the tested products, with a modest increase only after 24 h (T24), displaying significantly different values between the two fermentations (Figure 3B). The fermentation of both products promoted an increase in the butyric acid level only after 24 h (T24), and Microbiotal displayed a significantly higher amount, as compared to NBF 1 Figure 3C). 

## 4. Discussion

The microbial ecology of the canine large intestine still attracts great interest from the scientific community. One of the focusing topics is the effects of specific compounds or microorganisms, like probiotics and prebiotics, on the canine intestinal microbiota. This in vitro study evaluated the potentials of two commercial dietary supplements, Microbiotal cane^®^ and NBF 1^®^, to affect the canine intestinal environment in terms of microbiota composition and the production of SCFAs. 

The predominant phyla in the fermentation fluid consistent with previous reports in dogs included Firmicutes, Bacteroidetes, Fusobacteria, Proteobacteria, and Actinobacteria [19], and the results from NGS reported taxonomic profile variation trends within the two fermentations.

The results from enumeration by real-time PCR showed that the fermentation of both supplements can significantly increase the amount of *Lactobacillus* spp., *Bifidobacterium* spp., and *Bacteroides–Prevotella–Porphyromonas* spp., whereas the amount of the *Cl. coccoides*–*Eu. rectale* group remained quite stable during the fermentative process. *Staphylococcus* spp. displayed an increase at 24 h of the process only, whereas Enterobacteriaceae increased during the fermentation time. 

In detail, Microbiotal cane^®^ promoted a more immediate increase in *Lactobacillus* spp. after the first 6 h of fermentation, whereas NBF 1^®^ promoted the increase at the end of the process only. The two supplements supported an increase in the *Bifidobacterium* spp. counts only after 24 h. The fact that both *Lactobacillus* spp. and *Bifidobacterium* spp. increased in quantity during the fermentations of the tested supplements reveals the positive activities of Microbiotal cane^®^ and NBF 1^®^ on the gut canine microbiota, due to their capacity to increase bacterial groups involved in exerting beneficial effects on gut health. Like most intestinal bacteria, bifidobacteria are saccharolytic; they obtain carbon and energy through the fermentation of host and dietary carbohydrates. Bifidobacteria catabolize a variety of mono- and oligosaccharides released by glycosyl hydrolases acting on nondigestible plant polysaccharides or host-derived glycoproteins and glycoconjugates [20]. Microbiotal cane^®^ consists of fructo-oligosaccharides (FOS) and inulin that are mixtures of fructose moieties linked by glycosidic bonds with a terminal glucose unit [21]. FOS and inulin transit through the stomach and small intestine, where they are neither absorbed nor degraded, and reach the colon, where they are fermented by resident bacterial groups and promote the proliferation of bifidobacteria. Thus, FOS and inulin are effective prebiotics, defined as substrates, that are selectively utilized by host microorganisms, conferring a health benefit [22]. The fermentations of oligo- and polysaccharides in the colon are the result of intestinal microbial metabolic activity. During transit through the large bowel, unabsorbed carbohydrates, such as FOS and inulin, are hydrolyzed to their respective sugars; these sugars are fermented to SCFAs and biomass by the complex bacterial population. SCFAs represent the most important source of energy for colonocytes; they can stimulate the growth of colorectal mucosal cells, retard mucosal atrophy, and decrease the risk of malignant transformation in the colon. Butyrate has been shown to have anti-cancer effects both in in vitro cancer cell culture systems and in in vivo animal model experiments. The associated mechanisms involve many signaling pathways, as well as anti-inflammatory actions [23]. It stimulates mature colonocytes and inhibits undifferentiated malignant and stem cells [24].

Human in vivo trials have established that the addition of FOS or inulin to the diet leads to an increase in bifidobacteria [25], and several studies have described in vitro fermentation of FOS by human fecal cultures [26]. 

A different situation concerns *Bacteroides–Prevotella–Porphyromonas* spp., which increased during the fermentation of Microbiotal cane^®^, especially at the end of the process, whereas it remained quite stable at the end of NBF 1^®^’s fermentation, with respect to the starting point, even if an initial decrease was detected after 6 h. Among dominant beneficial bacteria, there are several species of *Bacteroides* that metabolize polysaccharides and oligosaccharides, providing nutrition and vitamins to the host and other intestinal microbial residents. The specific molecular interactions responsible for the beneficial and detrimental effects of *Bacteroides* species in humans [27] are not yet clear. It is already known that some pathogenic bacteria belong to this group, but current studies highlight that *Bacteroides–Prevotella–Porphyromonas* spp. also releases some beneficial metabolites, like SCFAs. Therefore, the fermentation of Microbiotal cane^®^ causing an increase in this bacterial group cannot be considered a negative result. 

The NGS data confirmed, in part, the modulation trend of the bacterial groups detected by real-time PCR. In addition, the presence of Fusobacteriaceae was highlighted with a notable existence of the *Fusobacterium* genus at 24 h in both fermentations. It is noteworthy that this bacterial group has a positive aspect, being prevalent in the guts of healthy dogs who typically spend a lot of time outside [28].

Both supplements exerted a stabilizing effect on the *Cl. coccoides–Eu. rectale* group, to which numerous harmful and pathogenic species belong. 

The Enterobacteriaceae family includes genera and species of prevalent fecal origin (*Escherichia*, *Salmonella*, and *Shigella*), environmental localization (*Budvicia*, *Buttiauxella*, *Citrobacter*, *Enterobacter*, *Erwinia*, *Klebsiella*, *Providencia*, *Serratia*, etc.), and mixed localization (*Citrobacter freundii*, *Enterobacter aerogenes,* and *E. cloacae*; *Klebsiella ozenae*, *K. oxytoca,* and *K. pneumoniae*; and *Proteus* spp.). Most of these bacterial species, which naturally inhabit human and animal intestines [29], have been detected in the two fermentation fluids by the NGS analysis.

The fermentation of the two supplements also promoted a non-significant increase in the *Staphylococcus* spp. after 6 h, becoming significant at the end of the process. Staphylococci are bacteria that commonly live on the skin, throat, and intestines of people and animals without causing problems; however, in particular conditions, they can penetrate the human/animal body and develop infections that are sometimes mild, other times serious. It is important to remember that the intestinal transit in dogs is much faster than in humans, and the intestinal fermentation usually resolves in the first 6–12 h. In the present study, what happens after 24 h of fermentation was also observed, but in dogs, such a long fermentation process never occurs. Consequently, the fact that staphylococci increased above all at the end of the 24 h of fermentation and not in the first 6 h can be considered a positive result. 

The acetic, propionic, and butyric acids were the only ones to increase after 24 h of fermentations and contributed to lowering the intestinal pH, reducing the growth of pathogenic species and influencing the intestinal microbiota composition [30]. Acetic acid derives from the metabolisms of *Bifidobacterium* spp. and *Lactobacillus* spp. [31]; the significant increase in this carboxylic acid at 24 h can be related to the increases in lactobacilli and bifidobacteria at that point. Among the metabolites released by the lactobacilli, butyric acid was also produced in a modest amount, especially at the end of the fermentation with Microbiotal cane^®^. On the other hand, butyric acid constitutes a major energy source for colonocytes, and it is partially metabolized by hepatocytes for gluconeogenesis with propionic acid [32]. The production of propionic acid was detected at a low level only after 24 h of fermentation for both supplements.

## 5. Conclusions

The in vitro fermentations of Microbiotal cane^®^ and NBF 1^®^ significantly modulate the dog intestinal microbiota. Microbiotal cane^®^ manages to have a short-term effect on increasing the concentration of lactobacilli; on the other hand, the administration of NBF 1^®^ may be of benefit in keeping under control the proliferation of harmful bacterial species. In case of alterations of the intestinal microbiota, the two products can be used selectively by stimulating and/or stabilizing the number of specific bacterial species.

Although in vitro assays facilitate experimentation, offering several advantages, caution must be taken in extrapolating results to in vivo conditions, as many variables and situations are implied.

## Data Availability

The authors will be available to share the data on demand.
